# Inuit Country Food and Health during Pregnancy and Early Childhood in the Circumpolar North: A Scoping Review

**DOI:** 10.3390/ijerph18052625

**Published:** 2021-03-05

**Authors:** Amy B. Caughey, Jan M. Sargeant, Helle Møller, Sherilee L. Harper

**Affiliations:** 1 Department of Population Medicine, University of Guelph, Guelph, ON N1G 2W1, Canada; sargeanj@uoguelph.ca (J.M.S.); sherilee.harper@ualberta.ca (S.L.H.); 2 Department of Health Sciences, Lakehead University, Thunder Bay, ON P7B 5E1, Canada; hmoeller@lakeheadu.ca; 3 School of Public Health, University of Alberta, Edmonton, AB T6G 2R3, Canada

**Keywords:** Inuit, nutrition, pregnancy, child, nutrition transition, food security, country food, traditional food, Circumpolar

## Abstract

Inuit communities in the Circumpolar North have experienced a nutrition transition characterized by the decreased intake of culturally important, nutrient-rich traditional food (country food), and an increased intake of market food, resulting in concerns over reduced diet quality and emerging chronic diseases. Nutrition in early life is critical for development, may influence health risks in later life, and is an important concern for Inuit community health. The goal of this scoping review was to characterize the nature, extent, and range of the published literature on Inuit country food and health in pregnancy and childhood. A search string was developed and applied to three databases, followed by title and abstract screening and full text review. Articles published between 1995 and 2019 were included, and data were extracted and summarized descriptively. The number of articles generally increased over time, despite the unequal geographic distribution of articles. The majority of the articles focused on environmental contaminants, and one-quarter described nutrient adequacy. Few articles described food security or food safety in pregnancy, and the most utilized quantitative methods. Gaps in understanding of country food use in pregnancy and early childhood highlight areas of future research to support public health policy for this population. Given the critical role of good nutrition in early life and the important contribution country food makes to diet quality for Inuit, further understanding of this interface is warranted.

## 1. Introduction

Over the past century, Indigenous Peoples around the world have undergone significant changes to food use and nutrition status, including Inuit communities in the Circumpolar North [1,2]. This nutrition transition has been described in Inuit communities and includes a decrease in the use of locally harvested traditional food (often termed country food, which includes locally harvested terrestrial and marine mammals, fish, shellfish, birds, and local plants and berries) and a parallel increase in the consumption of market food [3,4,5,6]. These changes have been associated with emerging chronic disease [7], including an increasing prevalence of childhood obesity [8], adult obesity, glucose intolerance, cardiovascular disease, and other diet-sensitive chronic conditions for Inuit living in Inuit communities in the circumpolar regions [9,10,11,12].

Along with this significant nutrition transition, food insecurity exists in many Inuit communities in the Circumpolar North [13,14,15,16,17,18,19]; being food insecure is a strong predictor of poorer mental and physical health, and has been associated with reduced diet quality, multiple indicators of chronic disease, and poor health outcomes for both adults and children [20,21]. Food insecurity among children is not only associated with adverse impacts to health and development, but also impacts school performance [22]. Indeed, nutrition in early human life, in particular the period of time from gestation through the first two years of life (1000 days), has long been regarded as a critical period when good nutrition can positively impact the nutrition and disease status through an individual’s life-cycle [23]. Links between the intrauterine environment and the development of chronic disease in later life, described as “developmental programming,” have been studied, and nutrition can impact the programming process [24]. The nutrition environment supported by maternal prenatal nutrition and early child nutrition, comprised of both adequate micro- and macronutrients (including protein, zinc, iron, folate, choline iodine, vitamins A, D, B_6,_ and B_12_) effects whether neurodevelopment occurs in a normal or abnormal way [25].

For Inuit communities, country food is a source of many nutrients that support good health, including protein, iron, zinc, vitamins A, D, B_12_, and n-3 fatty acids; country food is also important for cultural and spiritual well-being [6,26,27,28,29,30,31]. While the range of nutrients supplied by various Inuit country food has been described [32], considering the traditional (pre-colonial) Inuit diet is made up largely of animal-based foods, further work needs to advance global dietary guidelines to consider the principles of food sovereignty [33].

Dietary studies have reported that while country food contributed a low percentage to total energy intake, country food intake was associated with improved nutrient intake in adults [2,6,28,29,31,34,35]; furthermore, regular consumers of country food had higher intake of key micronutrients (including n-3 fatty acids, vitamins A, B_6_, B_12_, D, E) and lower intakes of carbohydrate, saturated fat, and sodium [36]. Country food has also been associated with a higher intake of protein and micronutrients, such as vitamin D and iron, that, when deficient, have been associated with conditions such as rickets and iron-deficiency anemia in Inuit children [37,38,39,40] highlighting the importance and potential of country food to help address nutrition-related health conditions among Inuit [40,41,42]. While the role of country food and impacts of nutrition transition on Inuit health has been explored [36], the state of research regarding the role of country food during pregnancy and early human life is less clear. Given that country food is a culturally appropriate and local food source for Inuit communities, and given the critical role of nutrition in pregnancy and early human life for immediate and long-term health, the objective of this scoping review was to systematically map published literature and explore the nature, extent, and range of research activity related to the role of country food in pregnancy and early childhood on health among Circumpolar Inuit.

## 2. Materials and Methods

### 2.1. Protocol

A scoping review approach was used to provide a systematic way of synthesizing knowledge to describe key concepts related to Inuit country food and health during pregnancy and early childhood, to identify gaps in knowledge, and to identify sources of evidence to inform research, practice, and policymaking [43,44]. A protocol for this scoping review was developed a priori and may be obtained from the primary author. The reporting of the methods and findings of this scoping review followed the checklist provided by Tricco and colleagues, and outlined in the Preferred Reporting Items for Systematic Reviews and Meta-Analyses Extension for Scoping Reviews (PRISMA-ScR) [45].

The protocol was developed within the context of the authors’ experience living, working in public health and researching within Inuit communities (AC, HM) and conducting health research with Inuit communities (SLH, JMS). Also, ongoing engagement and collaboration with Indigenous research partners and leaders, meetings with Inuit community health workers, and input from graduate students informed the protocol development, analysis and interpretation of this review and assisted with the contextualization of results.

### 2.2. Eligibility Criteria

The characteristics of the eligibility criteria used in this review are summarized in Table 1. The literature was restricted to the past twenty-five years, in order to provide insight into trends over time, while capturing the most current information on the topic. Because the focus of this review was pregnancy and early life, literature focusing on pregnant women and infants and children up to and including aged 10 years were included. As the review was in progress, it was clear that the age range selected during protocol development (children 0–18 years) frequency included dietary assessment of a primarily adult population, without results specific to child intake being reported. Therefore, to better capture nutrition in early life a decision was made to limit inclusion to children in the age range of 0–10 years old.

For this review, Inuit included Indigenous Peoples residing in Inuit homelands, and included Inupiat and Yupik (Alaska), Inuit and Inuvialuit (Canada), Kalaallit (Greenland), and Yupik (Chukotka, Russia), as defined by the Inuit Circumpolar Council (ICC)- [46]. Inuit have been living in the Arctic from time immemorial, and have been dependent on marine and terrestrial plants and animals of the Arctic Ocean, tundra and sea ice [47]. Demographic information for circumpolar Inuit regions is outlined in Table 2, and trends differ among Inuit regions; for instance, Nunavut and Alaska experience 3 to 4 times higher birth rates than Greenland where municipalities are seeing birthrate decline, and the population is older [48]. Canadian Inuit are a notably young population; 33% of Inuit in Canada are 14 years or younger [49]. Inuit also experience shorter life expectancy, most notable in Greenland and Canada where life expectancy for Inuit men and women is approximately 10 years below the national average [50]. Like many Indigenous peoples, Inuit have experienced a history of colonization and continue to experience colonialism and inequities in the social determinants of health [51]. Inuit communities have experienced rapid social and economic change, and an epidemiologic shift from infectious disease (such as tuberculosis–introduced with colonization) to chronic disease [52]; yet in many communities infectious disease remains a significant challenge [53].

In this scoping review, articles that focused on Inuit outside of Inuit homelands (e.g., in urban southern settings) were excluded. Articles were excluded if they focused only on store-bought food. Country food is described by Inuit as locally harvested foods (berries, narwhal, ringed seals, walrus, beluga whale, caribou, arctic char, polar bear, a variety of birds, etc.) and includes elements central to Inuit food and nutrition culture, such as the acts of harvesting, sharing, preparing and consuming these foods, as well as Inuit language to describe these practices and the passing on of associated traditions [54]. As such, to be included in this scoping review, the article had to mention country food in at least four sentences.

Reflecting Inuit conceptualizations of health [55,56], human health was defined holistically, and included components relevant to food-related health, such as breastfeeding and exposure to breastmilk, food security, and zoonotic food safety issues (Table 1). Articles that focused on the health of animals considered country food were included. Articles that focused on the health of wildlife that are not commonly considered country food, such as foxes and ravens, were excluded.

Primary research and cases studies were included in this review, reflecting the objective to map the extent, range, and nature of information contained in the published literature. Government and non-government agency reports, and other grey literature were excluded, given that the focus of this review was on published academic research.

### 2.3. Information Sources and Selection of Sources of Evidence

A search strategy was created to identify potentially relevant articles published between 1 January 1995 and 31 December 2019. The search string was developed to include Circumpolar Inuit, country food consumption, and health terms (Table 3). The search string was created in consultation with a research librarian at the University of Guelph. The databases Web of Science Core Collection, ™ CAB Direct, ^®^ and MEDLINE^®^ (Pubmed^®^) were searched without restriction on language or publication type. The search was conducted on 2 October 2018 and updated on 30 August 2020. Records were uploaded to Distiller SR© (Evidence Partners, Inc., Ottawa, ON, Canada) and deduplicated.

A two-step screening process was undertaken by two independent reviewers (Table 1). First, titles and abstracts were screened for relevance. There were no exclusions based on language. Articles about country food and a human health outcome in an Inuit population proceeded to the second step. In the second step, the full-text of potentially relevant articles were screened for date of publication (i.e., 1995–2019), population (i.e., Inuit), country food criteria, health criteria, and study design (Table 1). Two independent reviewers met to discuss and resolve conflicts, and when necessary, a third reviewer was involved.

### 2.4. Data Charting Process, Data Items, and Synthesis of Results

A form was created to identify study characteristics for extraction, including publication year, geographical scope of the study, study population, major theme of the study, and research methods employed. The information was extracted by one reviewer (AC) and verified by a review of ten percent by a second reviewer.

Data variables examined in this scoping review included year of publication, population (e.g., pregnant women, children up to and including age 10 years), major themes of the study (e.g., environmental contaminants, food security, diet and nutrition transition, nutrient deficiency, food security, food safety and zoonotic disease, and mental health), presence of community engagement, and research methods utilized (e.g., quantitative, qualitative, mixed methods). The data items were compiled and summarized in Microsoft Excel.

## 3. Results

**Figure 1:**
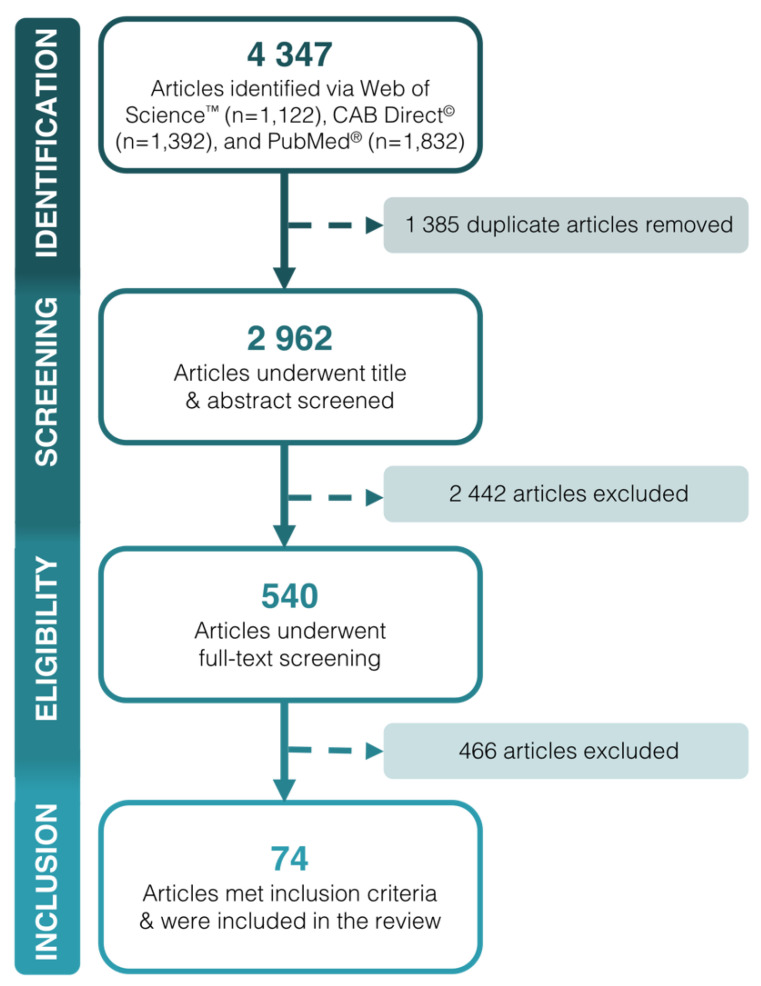
Flow diagram of search strategy to identify published articles about country food and Inuit health in pregnancy and early human life (1995–2019).

The search strategy identified 4347 citations from the three databases, with 2962 unique records identified after deduplication. A total of 74 articles met the inclusion criteria and were included in the scoping review (Figure 1). Full bibliographic details for included articles are included as Supplementary Materials. Articles that were excluded during full text screening did not include at least four sentences that discussed country food, were not primary research or cases studies, or were not inclusive of pregnant women or children up to and including ten years of age. The level of agreement for full text screening was calculated to be 96.3%.

### 3.1. Characteristics of Included Articles

**Figure 2:**
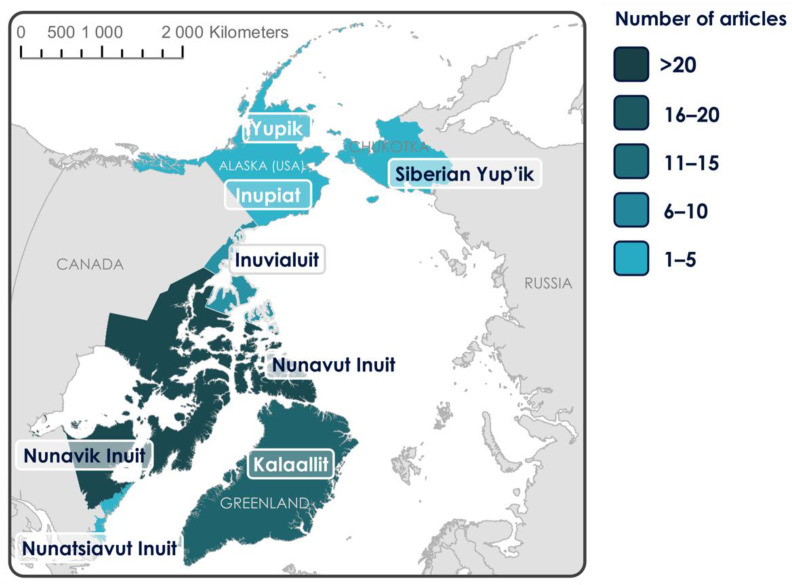
Heat map of primary research articles by location (*n* = 74) for Inuit country food and health in pregnancy and childhood by location in the Circumpolar North, described as member states by the Inuit Circumpolar Council, including the geographic regions of Greenland, Canada, USA, and Chukotka (Russia) [46].

The majority of primary research articles included in this scoping review were conducted in the Inuit regions of Nunavut (36%) and Nunavik (28%), Canada, followed by Kalaallit, Greenland (16%), the Inuvialuit Settlement Region (ISR), Canada (9%), and Nunatsiavut, Canada (5%) (Figure 2, created using Arc Map (version 10.7.1)). Three percent of articles conducted research in Yupik and Inupiat regions of Alaska, USA and 2% involved Siberian Yup’ik, Chukotka, Russia. Of note, in several instances one article reported on research conducted in more than one location, and therefore these categories were not mutually exclusive. There was an increase in published articles over time involving country food and health in pregnancy and early childhood, and the majority of articles were published between 2010 and 2019.

### 3.2. Research Themes and Population

**Figure 3:**
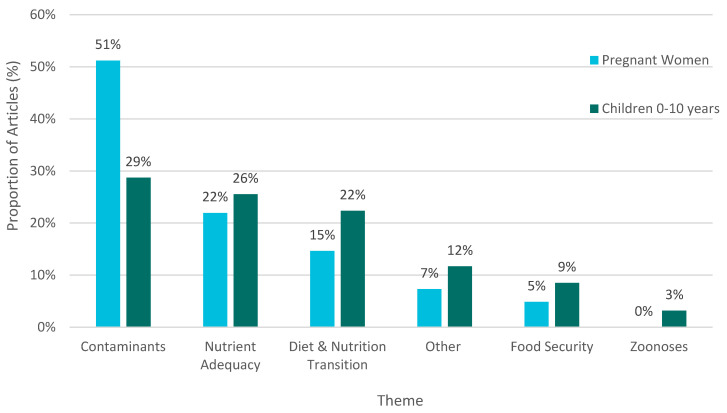
Study population represented by theme of primary research articles (n = 74) reporting on Inuit country food and health in pregnancy and early childhood (1995–2019).

For both pregnancy and childhood, the most common research theme was environmental contaminants, which made up over half of articles involving pregnant women (51%), and 29% of articles involving children (Figure 3). This area of research examined a range of topics, for example prenatal exposure to contaminants [57], monitoring of contaminants in umbilical cord blood [58], and mercury levels in the hair of children [59]. For all other themes in this review, there was a greater representation of children aged 0–10 years old than of pregnant women.

Nutrient adequacy (including micronutrient deficiency) was the next most common theme for both pregnant women and children, accounting for 22% of the articles involving pregnant women and 26% of the articles involving children. Examples of nutrients of focus in these articles included vitamin D levels in lactating women [60], vitamin D levels in preschool children [37], fatty acid levels in preschool children [61], and iron status of infants [62]. Articles also explored the relationship between intakes of traditional food and adequate nutrient intake for children [38,63]. Diet and the nutrition transition was a theme that represented 15% of articles involving pregnant women, including topics such as diet in pregnancy [64], while 22% of articles involving children in this theme explored emerging childhood obesity [10], traditional food intake of preschoolers [41], and oral health in children [65].

Food security was another theme described in this literature, and 5% of the articles with this theme involved pregnant women, while 9% of articles in the scoping review with a food security theme included children. These articles included characterization of food security prevalence [66] and correlates of food security [67] for Inuit preschoolers, whereas few articles reported on food security in pregnant women [68]. There were no articles discussing zoonoses and food safety as a major theme for pregnant women; 3% of articles with a zoonoses and food safety theme included children. The articles that included children were limited to cases studies of foodborne outbreaks occurring in Inuit communities related to botulism [69] and trichinellosis [70].

### 3.3. Geographic Distribution of Research Themes

**Figure 4:**
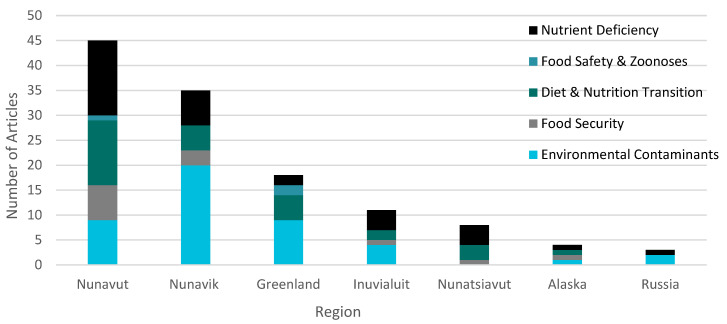
Geographic distribution of articles (n = 74) reporting on Inuit country food and health in pregnancy and childhood by major research theme (1995–2019).

The geographic distribution of articles by theme is outlined in Figure 4. Environmental contaminants were a prominent theme represented in all regions except Nunatsiavut; for instance, this theme was present in 72% of included articles from Nunavik and in 60% of included articles from Greenland. Articles in these regions tended to investigate contaminants of particular concern in pregnancy and childhood, including heavy metals [71], methylmercury [72], polychlorinated biphenyls [57,73] and lead [74].

The only theme that was present in all regions was related to nutrient deficiency; this theme was most prominent in the region of Nunavut with 44% of included articles having this focus. The articles focused on vitamin D [75,76], calcium [77], vitamin A [78], and iron [79] as micronutrients of study. Articles in Nunavik [41] and Nunavut [38] identified country food as an important contributor to nutrient intake in Inuit preschool children, and improved vitamin D status of Greenlandic schools was associated with consumption of marine mammals and fish [80].

Food safety and zoonoses as a theme was represented in two regions, Greenland and Nunavut, where 13% and 3% of articles included the theme, respectively. The articles were largely case studies of zoonotic disease outbreak situations [70]. Food security was present most often in articles in Nunavut where 21% of articles included food security as a theme, and Nunatsiavut where food security was a theme in 20% of articles. Other regions where food security was a theme in articles included Alaska (in 33% of articles), Inuvialuit Settlement Region (11%) and Nunavik (11%).

### 3.4. Research Methods

Most articles (91%) described quantitative methods (e.g., nutrition surveys, questionnaires, analysis of blood) as the primary method used. Qualitative research methods (7%) (e.g., interviews and focus groups), and mixed-method approaches (6%) were represented in few of the articles included in this scoping review. Quantitative research articles explored topics such as blood chemical concentrations attributed to traditional food consumption [81,82], breastfeeding duration rates [83,84], and diet in pregnancy [85], whereas qualitative research articles tended to address research questions related to the general experiences of pregnant women with pregnancy [86] or experiences of programs related to country food [68]. Of the quantitative articles included in this scoping review, 36% mentioned community engagement in the research. Qualitative research methods were only present in articles occurring in the regions of Nunavut (9%), Nunavik (7%), and the Inuvialuit region (11%). Mixed methods were employed in Nunavut (18%) and Nunavik (4%) regions.

## 4. Discussion

This scoping review utilized a systematic approach to explore the nature, extent and range of literature published over the past 25 years related to Inuit country food during pregnancy and early childhood and its immediate and life course impact on health. The volume of published research generally increased over the 25-year time period. The majority of the literature in this scoping review focused on the geographic regions of Nunavut and Nunavik, Canada, while Inuit regions in Alaska and Russia were the least represented.

The most common focus of articles was environmental contaminants, which represented over half of all articles involving pregnant women. Contaminant research was also the most represented theme in articles that included children. Research addressing the impacts of long-range contaminants (such as mercury) and local contaminant sources (such as lead shot) on the developing child [87,88,89,90,91] serves to inform risk-benefit conversations that consider the potential risk of exposure to contaminants alongside the benefit of high quality nutrition from, and cultural significance of, country food [92]. Outstanding questions are related to the interaction of environmental contaminants and nutrients, and how these may influence consumption guidance for Inuit communities [93,94].

While the safety of country food from the perspective of long-range contaminants was predominant in this scoping review, very few articles reported on zoonoses and food safety, and no articles involving pregnant women reported this theme. Certain zoonotic diseases, such as toxoplasmosis, present an important food safety concern for pregnant women and their fetuses. Acute infection with the toxoplasma parasite during pregnancy can introduce risk of transmission to the developing fetus [95], and congenital toxoplasmosis infection can result in stillbirth, miscarriage, and fetal deformities [96]. Such examples may be important considerations for Inuit communities since climate change stands to increase the risk of zoonotic and foodborne diseases in the Arctic [97]. As such, there is a need to understand the impacts of both environmental contaminant and zoonotic and foodborne disease risk for vulnerable populations, including pregnant women and children.

Of interest, this scoping review did not identify articles that looked across themes related to zoonotic disease and environmental contaminants, which are both intimately connected to wildlife and human health in Inuit communities. A multidisciplinary approach that describes how the health of pregnant women is impacted by these together— zoonotic disease, contaminants, and climate change—would support an Inuit directed priority setting [98] as well as priorities of academic entities [97] and could provide a useful framework on which to support public health guidance for pregnant women in Inuit communities.

While it is important to consider potential risks introduced to Inuit by contaminants, zoonotic disease and other food safety issues, there is also a need to understand the consequences that may result from any decreased intake of country food [99] and subsequent impacts on nutrition status and health for Inuit women and their children. Compromised nutrition during pregnancy and infancy is a serious public health issue [37,100]; failure to provide key nutrients during this critical period of development may result not only in lifelong impacts on chronic disease risk but also in deficits in brain development, which cannot be corrected by nutrient repletion [25].

In this review, less than one-quarter of the articles focused on nutrient adequacy or micronutrient deficiency. Inuit communities experience nutrient deficiencies in both women and children [37,101,102,103,104] that impact the overall health and development over the life course [79] and impact risk of infectious illness for both children [105] and adults [106]. It is well-established that Inuit country food is rich in a variety of macro and micronutrients [32], including nutrients of particular concern in the Circumpolar North such as vitamin D [39]. In Alaska, serum vitamin D decline was associated with a decline in consumption of traditional marine food among young women [102]. Marine mammals and fish consumption was also associated with positive vitamin D status of children in Greenland, despite a high prevalence of vitamin D deficiency in children [80]. Study of preschool children in Nunavut, Canada also revealed high vitamin D deficiency, and found improved vitamin D status with higher intakes of vitamin D fortified milk, but also suggested that improved intake of traditional foods rich in vitamin D could improve vitamin D status of preschool children [37]. The role of traditional food in addressing nutrient adequacy and micronutrient deficiency may be an area deserving of further consideration [30]. Inuit representative organizations have identified that country food is a preferred food for Inuit communities, while acknowledging that work remains to ensure these are accessible for Inuit communities [54].

Indeed, this scoping review revealed a gap in understanding of the barriers that pregnant women and their children experience in accessing, and ultimately consuming, country food. Only 5% of the articles involving pregnant women had food security as a major theme, and only 9% of articles involving children described food security in relation to children. Food insecurity is strongly associated with poor nutrition and adverse physical and mental health outcomes, and this applies across the lifecycle [107]. The Nunavut Inuit Child Health Survey (2007–08) identified nearly 70% food insecurity amongst households with preschool children [9,66]. In Greenland, school aged children have been the focus of food insecurity research, with both Canada and Greenland identifying that food insecurity among children is a critical public health issue [9,21,22,72] requiring further attention.

Food insecurity is a significant issue in Inuit communities [41], yet little has been published about the factors that influence food insecurity for Inuit women and children. Research has described gendered dimensions of food security among Inuit women and coping mechanisms for food insecurity that include both compromising nutrient intake and sharing country food within the community [108]. Having a hunter in a home has been associated with improved food security for Inuit women of childbearing age in Nunavut [39,109], yet food security experiences of pregnant women are less clear. An enhancing understanding of experiences related to country food access, availability and acceptance in pregnancy may better inform how to best support the nutrition needs of pregnant women.

Breastfeeding in the context of infant food security was represented in this review, and breastfeeding is a culturally grounded mechanism of food security for infants in Inuit communities, with the potential to improve health outcomes for women and children [83,110]. Understanding the role of country food in supporting healthy pregnancy and lactation is an important research gap that with increased attention could help inform best-practices for supporting families experiencing food insecurity.

The research methods used in the research were explored in this scoping review. The vast majority of articles included in this scoping review utilized quantitative methods. Quantitative methods based research, while providing robust and objective information, is less likely to report community engagement [111]; in this scoping review, just over one-third of quantitative focused research articles reported community engagement.

While quantitative methods predominate health research, qualitative data collection methods provide validated and comparable methods to assess dietary intake, dietary adequacy, nutrient intake, and describe risk of disease [112]. Qualitative methods based research is well aligned with Indigenous methodologies and methods described worldwide [112]. Inuit ways of knowing and sharing information such as storytelling and oral history are culturally appropriate research methods [113,114,115] that support decolonizing approaches to research called for by Inuit organizations [115]. The decolonization of research does not mean rejecting research or Western knowledge, but rather calls for the methodologies and methods of research to be decolonized [112].

Further, research centered on qualitative methods has provided rich information for Inuit communities around issues critical to health and well-being including country food, harvesting, mental health and a changing climate [113]. Qualitative research is concerned with understanding how people make sense of their world, and the meaning of experiences [116]; advancing engagement in qualitative research to inform public health practice may help in understanding the full meaning of the primarily quantitative research described in this review [116].

The goal of this review was to examine published academic literature; as such, this review did not include graduate theses or government and non-government agency reports which can be rich sources of information related to country food and may also include Inuit community and Inuit representative organization perspectives and priorities [56,103,115]. Therefore, this review could have excluded ideas, themes, and priorities that may not have been captured in peer-reviewed journals.

## 5. Conclusions

This scoping review of country food and health in pregnancy and childhood in Inuit communities in the Circumpolar north revealed that the number of published articles on this topic have increased over the past quarter century. The majority of the research involving pregnant women and children related to environmental contaminants across the circumpolar north, and only one-quarter of research included discussion of nutrient adequacy or micronutrient deficiency, even though these challenges exist and remain issues of vital public health consequence for Inuit during pregnancy and childhood and may influence disease in later life. Few articles identified food security, food safety and zoonotic disease as major themes, even though these have the potential to impact the perinatal period and hold significant influence on nutrition in early life. The research was largely focused on quantitative data collection methods, with few articles using qualitative methods despite these being well-aligned with Inuit knowledge sharing practices and decolonizing approaches to health research. A further exploration of country food in pregnancy and in childhood serves not only to support public health policy and healthcare service delivery in Inuit communities, but also may enrich the understanding of food sovereignty and the nutrition transition in this critical period of the lifespan, and beyond.

## Figures and Tables

**Table 1:** Summary of inclusion and exclusion criteria that were used to identify Inuit country food and health in pregnancy and childhood literature (1995–2019). “X” indicates criterion was used at each respective level of screening.

Criteria	Study Characteristic	Level 1 Screen: Title & Abstract	Level 2 Screen: Full Text
Date	Inclusion:		
	Date of publication was 1995 or later		X
Population	Inclusion:		
	Inuit as defined by Inuit Circumpolar Council (ICC)	X	X
	Pregnant women, cord blood, children 0–10 years		X
	Exclusion:		
	Indigenous groups not meeting ICC definition of Inuit	X	X
	Inuit living outside of Inuit homelands (e.g., in southern urban settings)	X	X
	Non-pregnant women, children age >10 years		X
Food	Inclusion:		
	Country food discussed in at least 4 sentences		X
	Exclusion:		
	Assessment only on retail food (e.g., store-bought food)	X	X
	Focus on animals not considered food source (e.g., fox, raven)	X	X
Human Health	Inclusion:		
	Any human health, wellness, nutrition, syndrome, and/or disease outcome (including breastfeeding, food security)	X	X
	Animal health of food source animals	X	X
	Exclusion:		
	Focus on animal health of non-food source animal (e.g., fox, raven)	X	X
Study Design	Inclusion:		
	Primary research study, including case studies		X
	Exclusion:		
	PhD thesis, government reports, conference proceedings		X

**Table 2:** Demographic information for circumpolar Inuit regions of Alaska, Canada, Greenland, and Russia (Chukotka) [50,51,52,53,54,55,56].

	Alaska	Canada	Greenland	Chukotka
Approximate Inuit population size	48,300	47,000 Nunavut: 30,000 Nunavik: 11,800 Nunatsiavut: 2285 Inuvialuit: 3100	51,300	1600
Birth rate in Inuit Region	High	High	Declining	n/a
Inuit infant mortality per 1000 live births	10.8	15.3 (Nunavut)	12.1	20.3
National infant mortality per 1000 live births	6.9 (US)	5.3 (Canada)	4.7 (Denmark)	13.3 (Russia)

**Table 3:** Summary of the search string that was developed to search databases for research about Circumpolar Inuit, country food, and human health topics (1995–2019). Asterisk (*) represents wildcard symbol used to capture other words beginning with the same letters.

Search String Components	Search String
Circumpolar Inuit **	Circumpolar OR Inuit* OR Inuvialuit OR Inupiat OR Yupik OR Yup’ik OR Eskimo* OR Aleut* OR arctic OR subarctic OR “Alaskan native” OR “Northern Canada” OR Nunangat OR Kalaallit OR Nunavut OR Greenland OR Nunatsiavut OR Inuvialuit OR Nunavik OR NunatuKavut OR Chukchi
Country food consumption	food OR diet
Human health	health OR disease* OR infect* OR syndrom* OR nutrition OR malnutrition OR wellness OR wellbeing
Final Search String used in CAB Direct ^®^	(Circumpolar OR Inuit* OR Inuvialuit OR Inupiat OR Yupik OR Yup’ik OR Eskimo* OR Aleut* OR arctic OR subarctic OR “Alaskan native” OR “Northern Canada” OR Nunangat OR Kalaallit OR Nunavut OR Greenland OR Nunatsiavut OR Inuvialuit OR Nunavik OR NunatuKavut OR Chukchi) AND (food OR diet) AND (health OR disease* OR infect* OR syndrom* OR nutrition OR malnutrition OR wellness OR wellbeing)

** Terms used to identify Circumpolar Inuit were based on both umbrella terms for Inuit as well as individual group names. Note, to develop a search string that would capture all research that is potentially relevant to Inuit, the authors also had to acknowledge a long history of unethical and harmful research that has been conducted on Inuit, and associated terminology used in reference to them. As such, this search string includes terms that are offensive and have been historically used to marginalize Inuit. The use of these terms in no way reflect the authors’ beliefs or relations with Inuit. Additionally, all articles generated by this search string were evaluated to ensure that no racist or eugenic research was included in this review.

## Data Availability

Please see Supplementary Materials for the articles (i.e., “data”) included in the review.

## Supplementary Materials

The following are available online at https://www.mdpi.com/1660-4601/18/5/2625/s1, Table S1: Articles included in the review “Inuit country food and health during pregnancy and early childhood in the Circumpolar North: A scoping review”.
