# Supplementary material for: Inuit Country Food and Health during Pregnancy and Early Childhood in the Circumpolar North: A Scoping Review
Source: Int J Environ Res Public Health. 2021 Mar 5;18(5):2625. doi: 10.3390/ijerph18052625 (PMC7967653; doi:10.3390/ijerph18052625)
Supplement: Supplementary file 1 [file ijerph-18-02625-s001.pdf]

Table S1. Articles included in the review "Inuit country food and health during pregnancy and early childhood in the Circumpolar North: a scoping review".

Reference	Publication Year	Region	Theme
Chan et al. (1995) Assessment of dietary exposure to trace metals in Baffin Inuit food. Environmental Health Perspectives, 103(7-8): 740	1995	Nunavut	Contaminants
Young (1995) The population survey as a tool for assessing family health in the Keewatin region, NWT, Canada. Arctic Medical Research, 54 Suppl 1: 77	1995	Nunavut	Diet & Nutrition<br>Nutrient Adequacy
Kuhnlein et al. (1996) Dietary nutrient profiles of Canadian Baffin Island Inuit differ by food source, season, and age. Journal of the American Dietetic Association, 96(2): 155	1996	Nunavut	Diet & Nutrition<br>Nutrient Adequacy
Lawn et al. (1998) Food consumption patterns of Inuit women. International Journal of Circumpolar Health, 57 Suppl 1: 198	1998	Nunavut<br>Nunatsiavut	Diet & Nutrition<br>Nutrient Adequacy
G Muckle, E Dewailly, P Ayotte (1998). Prenatal exposure of Canadian children to polychlorinated biphenyls and mercury. Canadian Journal of Public Health, 89 Suppl 1: S20	1998	Nunavut<br>Nunavik<br>Inuvialuit	Contaminants
Deutch et al. (1999) High blood levels of persistent organic pollutants are statistically correlated with smoking. International Journal of Circumpolar Health, 58(3): 214	1999	Greenland	Contaminants
Waiters et al. (1999) Perinatal vitamin D and calcium status of northern Canadian mothers and their newborn infants. Journal of the American College of Nutrition, 18(2): 122	1998	Inuvialuit	Nutrient Adequacy
Berti et al. (1999) Food use and nutrient adequacy in Baffin Inuit children and adolescents. Canadian Journal of Dietetic Practice and Research, 60(2): 63	1999	Nunavut	Diet & Nutrition<br>Nutrient Adequacy
Deutch et al. (2000) High human plasma levels of organochlorine compounds in Greenland. Regional differences and lifestyle effects. Danish Medical Bulletin, 47(2): 132	2000	Greenland	Contaminants
Bjerregaard et al. (2000) Organochlorines and heavy metals in pregnant women from the Disko Bay area in Greenland. Science of the Total Environment, 245(1/3): 195	2000	Greenland	Contaminants
Willows et al. (2000) Anemia and iron status in Inuit infants from northern Quebec. Canadian Journal of Public Health, 91(6): 407	2000	Nunavik	Nutrient Adequacy

Serhir et al. (2001) Outbreak of trichinellosis associated with arctic walruses in Northern Canada, 1999. Canada Communicable Disease Report, 27(4): 31	2001	Nunavut	Zoonoses
Muckle et al. (2001) Determinants of polychlorinated biphenyls and methylmercury exposure in Inuit women of childbearing age. Environmental Health Perspectives, 109(9): 957	2001	Nunavik	Contaminants
Muckle et al. (2001) Prenatal exposure of the Northern Québec Inuit infants to environmental contaminants. Environmental Health Perspectives, 109(12): 1291	2001	Nunavik	Contaminants
Levesque et al. (2003) Monitoring of umbilical cord blood lead levels and sources assessment among the Inuit. Occupational and Environmental Medicine, 60(9): 693	2003	Nunavik	Contaminants
Dallaire et al. (2003) Time trends of persistent organic pollutants and heavy metals in umbilical cord blood of Inuit infants born in Nunavik (Québec, Canada) between 1994 and 2001. Environmental Health Perspectives, 111(13): 1660	2003	Nunavik	Contaminants
Ayotte et al. (2003) Assessment of pre- and postnatal exposure to polychlorinated biphenyls: lessons from the Inuit cohort study. Environmental Health Perspectives, 111(9): 1253	2003	Nunavik	Contaminants
Dudarev et al. (2004) Blood concentrations of persistent toxic substances in the Indigenous communities of the Russian Arctic. International Journal of Circumpolar Health, 63 Suppl 2, 179	2003	Russia	Contaminants
Arbour et al. (2004) Heart defects and other malformations in the Inuit in Canada: a baseline study. International Journal of Circumpolar Health, 63(3): 251	2004	Nunavut<br>Nunavik	Nutrient Adequacy
Van Oostdam et al. (2004) Circumpolar maternal blood contaminant survey, 1994-1997 organochlorine compounds. Science of the Total Environment, 330(1-3): 55	2004	Greenland<br>Nunavut<br>Nunavik<br>Alaska<br>Russia	Contaminants
Lucas et al. (2004) Gestational age and birth weight in relation to n-3 fatty acids among Inuit (Canada). Lipids, 39(7): 617	2004	Nunavik	Contaminants<br>Nutrient Adequacy
Christofides et al. (2005) Iron deficiency and anemia prevalence and associated etiologic risk factors in First Nations and Inuit communities in	2005	Nunavut	Diet & Nutrition<br>Nutrient Adequacy

Northern Ontario and Nunavut. Canadian Journal of Public Health, 96(4): 304			
Møller et al. (2005) Outbreak of trichinellosis associated with consumption of game meat in West Greenland. Veterinary Parasitology, 132(1/2): 131	2005	Greenland	Zoonoses
Saint-Amour et al. (2006) Alterations of visual evoked potentials in preschool Inuit children exposed to methylmercury and polychlorinated biphenyls from a marine diet. Neurotoxicology, 27(4): 567	2006	Nunavik	Contaminants
Butler Walker et al. (2006) Maternal and umbilical cord blood levels of mercury, lead, cadmium, and essential trace elements in Arctic Canada. Environmental Research, 100(3): 295	2006	Nunavut Inuvialuit	Contaminants
Berti et al. (2008) Dietary assessment of Indigenous Canadian Arctic women with a focus on pregnancy and lactation. International Journal of Circumpolar Health, 67(4): 349	2008	Nunavut Nunatsiavut Inuvialuit	Diet & Nutrition Nutrient Adequacy
Gessner (2009) Geographic and racial patterns of anemia prevalence among low-income Alaskan children and pregnant or postpartum women limit potential etiologies. Journal of Pediatric Gastroenterology and Nutrition, 48(4): 475	2009	Alaska	Nutrient Adequacy
El Hayek et al. (2010) Vitamin D status of Inuit preschoolers reflects season and vitamin D intake. The Journal of Nutrition, 140(10): 1839	2010	Nunavut	Nutrient Adequacy
Galloway et al. (2010) Emerging obesity among preschool-aged Canadian Inuit children: results from the Nunavut Inuit Child Health Survey. International Journal of Circumpolar Health, 69(2): 151	2010	Nunavut	Diet & Nutrition
Boucher et al (2010) Prenatal exposure to methylmercury and PCBs affects distinct stages of information processing: an event-related potential study with Inuit children. Neurotoxicology, 31(4): 373	2010	Nunavik	Contaminants
Egeland et al. (2010) Food insecurity among Inuit preschoolers: Nunavut Inuit Child Health Survey, 2007-2008. Canadian Medical Association Journal, 182(3): 243	2010	Nunavut	Food Security
Egeland et al. (2010) Cultural, socioeconomic, and health indicators among Inuit preschoolers: Nunavut Inuit Child Health Survey, 2007-2008. Rural and Remote Health, 10(2): 1365	2010	Nunavut	Food Security Diet & Nutrition

Pacey et al. (2010) Prevalence and risk factors for parental-reported oral health of Inuit preschoolers: Nunavut Inuit Child Health Survey, 2007-2008. Rural and Remote Health, 10(2): 1368	2010	Nunavut	Diet & Nutrition<br>Nutrient Adequacy
Johnson-Down et al. (2010) Adequate nutrient intakes are associated with traditional food consumption in Nunavut Inuit children aged 3-5 years. Journal of Nutrition, 140(7): 1311	2010	Nunavut	Diet & Nutrition<br>Nutrient Adequacy
El-Hayek et al. (2010) Vitamin D status of Inuit preschoolers reflects season and vitamin D intake. Journal of Nutrition, 140(10): 1839	2010	Nunavut	Nutrient Adequacy
Egeland et al. (2011) Traditional food and monetary access to market-food: correlates of food insecurity among Inuit preschoolers. International Journal of Circumpolar Health, 70(4): 373	2011	Nunavut	Food Security<br>Nutrient Adequacy
Boucher (2011) Neurophysiologic and neurobehavioral evidence of beneficial effects of prenatal omega-3 fatty acid intake on memory function at school age. American Journal of Clinical Nutrition, 93(5): 1025	2011	Nunavik	Contaminants
WenJing et al. (2011) Mercury hair concentrations and dietary exposure among Inuit preschool children in Nunavut, Canada. Environment International, 37(1): 42	2011	Nunavut	Contaminants
Ethier et al. (2012) Effects of environmental contaminant exposure on visual brain development: a prospective electrophysiological study in school-aged children. Neurotoxicology, 33(5): 1075	2012	Nunavik	Contaminants
El Hayek et al. (2012) Higher body mass, older age and higher monounsaturated fatty acids intake reflect better quantitative ultrasound parameters in Inuit preschoolers International Journal of Circumpolar Health, 71	2012	Nunavut	Nutrient Adequacy
Gagne et al. (2012) Traditional food consumption is associated with higher nutrient intakes in Inuit children attending childcare centres in Nunavik. International Journal of Circumpolar Health, 71(1): 18401	2012	Nunavik	Diet & Nutrition<br>Nutrient Adequacy
Boucher et al. (2012) Response inhibition and error monitoring during a visual go/no-go task in Inuit children exposed to lead, polychlorinated biphenyls, and methylmercury. Environmental Health Perspectives, 120(4): 608	2012	Nunavik	Contaminants
Boucher et al. (2012) Prenatal methylmercury, postnatal lead exposure, and evidence of attention deficit/hyperactivity disorder among Inuit	2012	Nunavik	Contaminants

children in Arctic Québec. Environmental Health Perspectives, 120(10): 1456			
Langlois et al. (2013) Dietary habits of Aboriginal children. Health reports, 24(4): 3	2013	Nunavut<br>Nunavik<br>Nunatsiavut<br>Inuvialuit	Diet & Nutrition<br>Nutrient Adequacy
Findlay et al. (2013) Hunger among Inuit children in Canada. International Journal of Circumpolar Health, 72	2013	Nunavut<br>Nunavik<br>Nunatsiavut<br>Inuvialuit	Food Security
Dallaire et al. (2013) Exposure to organochlorines and mercury through fish and marine mammal consumption: associations with growth and duration of gestation among Inuit newborns. Environment International, 54, 85	2013	Nunavik	Contaminants
Gagne et al. (2013) Consumption of tomato products is associated with lower blood mercury levels in Inuit preschool children. Food and Chemical Toxicology, 51: 404	2013	Nunavik	Contaminants
Jensen et al. (2013) Tobacco smoke increases the risk of otitis media among Greenlandic Inuit children while exposure to organochlorines remain insignificant. Environment International, 54: 112	2013	Greenland	Contaminants
Blanchet et al. (2014) Usual dietary fatty acid intakes and red-blood-cell membrane fatty acid composition in Inuit children attending child-care centres in Nunavik, northern Québec, Canada. Public Health Nutrition, 17(12): 2844	2014	Nunavik	Diet & Nutrition<br>Nutrient Adequacy
McIsaac et al. (2014) Exclusive breastfeeding among Canadian Inuit results from the Nunavut Inuit Child Health Survey. Journal of Human Lactation, 30(2): 229	2014	Nunavut	Diet & Nutrition
Fillion et al. (2014) Identification of environmental sources of lead exposure in Nunavut (Canada) using stable isotope analyses. Environment International, 71, 63	2014	Nunavut	Contaminants
Curren et al. (2014) Comparing plasma concentrations of persistent organic pollutants and metals in primiparous women from northern and southern Canada. Science of the Total Environment, 306	2014	Nunavut	Contaminants

O'Brien et al. (2014) Effect of dietary calcium intake on lead exposure in Inuit children attending childcare centres in Nunavik. International Journal of Environmental Health Research, 24(5): 482	2014	Nunavik	Contaminants<br>Diet & Nutrition<br>Nutrient Adequacy
Macdonald et al. (2014) Understanding healthy pregnancies: the perspective of Inuit midwives in northwestern Quebec. Canadian Journal of Rural Medicine, 19(4): 128	2014	Nunavik	Food Security<br>Diet & Nutrition
Singer et al. (2014) Food consumption, obesity and abnormal glycaemic control in a Canadian Inuit community. Clinical Obesity, 4(6): 316	2014	Nunavut	Diet & Nutrition<br>Nutrient Adequacy
McIsaac et al. (2015) Prevalence and Characteristics Associated with Breastfeeding Initiation Among Canadian Inuit from the 2007-2008 Nunavut Inuit Child Health Survey. Maternal and Child Health Journal, 19(9): 2003	2015	Nunavut	Food Security<br>Diet & Nutrition
Hedinsdottir Hammer et al. (2015). Fatal outbreak of botulism in Greenland. Infectious Diseases, 47(3): 190	2015	Greenland	Zoonoses
Curren et al. (2015) Assessing determinants of maternal blood concentrations for persistent organic pollutants and metals in the eastern and western Canadian Arctic. Science of the Total Environment, 150	2015	Nunavut<br>Inuvialuit	Contaminants
Knudsen et al (2015) Lifestyle, reproductive factors and food intake in Greenlandic pregnant women: the ACCEPT - sub-study. International Journal of Circumpolar Health, 74, 29469	2015	Greenland	Diet & Nutrition<br>Nutrient Adequacy
Long (2015) Food intake and serum persistent organic pollutants in the Greenlandic pregnant women: the ACCEPT sub-study. Science of the Total Environment, 529, 198	2015	Greenland	Contaminants
Binnington et al. (2016). Mechanistic polychlorinated biphenyl exposure modeling of mothers in the Canadian Arctic: the challenge of reliably establishing dietary composition. Environment International, 92-93, 256	2016	Nunavut<br>Inuvialuit	Contaminants
Gautier et al. (2016) Assessment of the implementation fidelity of the Arctic Char Distribution Project in Nunavik, Quebec. BMJ Global Health, 1(3): e000093	2016	Nunavik	Contaminants<br>Food Security<br>Nutrient Adequacy
Maurice et al. (2017) Yup'ik identity and socioeconomic status are associated with child consumption of traditional food and weight in rural Yup'ik communities. Ethnicity & Health, 1-11	2017	Alaska	Food Security<br>Diet & Nutrition
McIsaac et al. (2017) Household food security and breast-feeding duration among Canadian Inuit. Public Health Nutrition, 20(1): 64	2017	Nunavut	Food Security

Huet et al. (2017) Food insecurity and food consumption by season in households with children in an Arctic city: a cross-sectional study. BMC Public Health, 17(578)	2017	Nunavut	Food Security
Terkelsen (2018) Reproductive factors, lifestyle and dietary habits among pregnant women in Greenland: The ACCEPT sub-study 2013-2015. Scandinavian Journal of Public Health, 46(2): 252	2018	Greenland	Diet & Nutrition<br>Nutrient Adequacy
Rasmussen et al. (2019) Greenlandic women's lifestyle and diet during pregnancy and child risk for asthma, eczema and allergy: an ACCEPT-sub-study. International Journal of Circumpolar Health, 78(1): 1682421	2019	Greenland	Contaminants<br>Diet & Nutrition
Rasmussen et al. (2019) Greenlandic women´s lifestyle and diet during pregnancy and child risk for asthma, eczema and allergy: an ACCEPT-sub-study. International Journal of Circumpolar Health, 78(1): 1682421	2019	Greenland	Diet & Nutrition
Timmermann et al. (2019) Environmental chemical exposures among Greenlandic children in relation to diet and residence. International Journal of Circumpolar Health, 78(1): 1642090	2019	Greenland	Contaminants
Courraud et al. (2019) Dietary habits, metabolic health and vitamin D status in Greenlandic children. Public Health Nutrition, 23(5): 904	2019	Greenland	Diet & Nutrition<br>Nutrient Adequacy
Bank-Nielsen et al. (2019) Pregnant Inuit Women's Exposure to Metals and Association with Fetal Growth Outcomes: ACCEPT 2010-2015. International Journal of Environmental Research and Public Health, 16(7)	2019	Greenland	Contaminants
Adamou et al. (2018) Socio-economic inequalities in blood mercury (Hg) and serum polychlorinated biphenyl (PCB) concentrations among pregnant Inuit women from Nunavik, Canada. Canadian Journal of Public Health, 109(5-6): 671	2018	Nunavik	Contaminants
Turgeon et al. (2019) Temporal trends of legacy and emerging persistent organic pollutants in Inuit preschoolers from Northern Quebec (Canada). International Journal of Environmental Health Research, 29(6): 643	2019	Nunavik	Contaminants
El Hayek et al. (2018) Vitamin D status and intake of lactating Inuit women living in the Canadian Arctic. Public Health Nutrition, 21(11): 1988	2018	Nunavut<br>Nunatsiavut<br>Inuvialuit	Nutrient Adequacy
